# 

**Published:** 1877-03-20

**Authors:** 


					﻿,S
W
fl
w
a
o
>—<
fl
w
p
o
o
H
A
«
P
O
>-j
a?
H
w
E-l
fl
o
•QQ
•—I
<1
fl
Q
fl
w
H
fl
fl
GO
fl
«
fl
fl
GO
fl
fl
fl
VOL. VH. w	MARCH 20, 1877.	No. 8.
THE
SOUTHERN MEDICAL RECORD.
A MONTHLY JOURNAL OF PRACTICAL MEDICINE.
« QUJCQUID PRIECIPIES ESTO BREVIS.99
a>
N
o
B
H
O
O
§
q
g
g
H
i—i
O
QD
>
d
d
B
Q
A
Al
Q
d
M
A
B
g
At
OQ
CD
o
d
£
►4
fl
B
d
JAS^P. HARRISON & CO., Printers.
ATLANTA, GEORGIA.
TERMS: $2.00 per Annum in Advance—Club Rates, 5 Copips for $8.00.
				

